# The Post-illumination Pupil Response (PIPR) Is Associated With Cognitive Function in an Epidemiologic Cohort Study

**DOI:** 10.3389/fneur.2019.00682

**Published:** 2019-06-26

**Authors:** Yanjun Chen, Alex A. Pinto, Adam J. Paulsen, Carla R. Schubert, Laura M. Hancock, Barbara E. Klein, Ron Klein, Karen J. Cruickshanks

**Affiliations:** ^1^Department of Ophthalmology and Visual Sciences, University of Wisconsin School of Medicine and Public Health, Madison, WI, United States; ^2^Department of Neurology, University of Wisconsin School of Medicine and Public Health, Madison, WI, United States; ^3^Department of Population Health Sciences, University of Wisconsin School of Medicine and Public Health, Madison, WI, United States

**Keywords:** pupillography, retinal ganglion cells, melanopsin, cognition, aging

## Abstract

We conducted a cross-sectional study on 403 participants in the 10-year follow-up examination of the Beaver Dam Offspring Study. The participants included 172 male and 231 female, with age ranging from 33 to 81 years (mean ± SD, 60.7 ± 9.3). The post-illumination pupil response (PIPR) was recorded using binocular infrared pupillometer (Neur-Optics, Inc., Irvine, CA). Cognitive testing consisted of Trail Making Test (TMT) Parts A and B, Rey Auditory Verbal Learning Test (AVLT), Digit Symbol Substitution Test (DSST), and Verbal Fluency Test (VFT) (F, A, and S). Principal component analysis (PCA) was used to calculate an overall cognitive function score. There was a significant reduction in the mean baseline pupil diameter by 0.21 mm for every 5-year increase in age (95% CI: −0.25, −0.17). There was also a significant increase in the PCA cognitive score by 0.20 (linear regression, 95% CI: 0.08, 0.32) for every 0.1 unit increase in the PIPR. The association remained significant after adjusting for age, sex, education, medications, systemic and ocular disease, and short form-12 physical and mental component score. The results of this study demonstrated a modest association between the PIPR and cognitive function, warranting longitudinal studies to evaluate the role of the PIPR in predicting cognitive function in the middle-aged and older adults.

## Introduction

Whereas visual impairment has been associated with increased morbidity and mortality in a variety of age-related ocular diseases, studies have also shown that age-related ocular diseases are associated with declines in quality-of-life measures independent of vision (1, 2). However, how the visual system—from the eyes to the brain—works to influence behavior and quality of life remains unclear. The recent discovery of the intrinsically photosensitive retinal ganglion cells (ipRGCs), a group of novel, melanopsin-containing photoreceptors in the inner retina that connect the eye to the brain, provides us a unique opportunity to understand how the eye's modulation of external ambient light impacts brain activity (3, 4).

The eye serves two crucial physiological functions. In addition to its well-known functionality of producing vision (image-forming function), the eye also plays a critical role in conducting a collection of vital, non-visual physiologic activities including circadian rhythm, pineal melatonin suppression, rest-activity, body temperature, and hormonal secretion (5). The non-image-forming function of the eye relies on the direct connection between the ipRGCs and the suprachiasmatic nucleus of the hypothalamus, the master biological clock that synchronizes the majority of biological and physiological activities in living organisms with the external 24-h light-dark cycle (6, 7). When experimental animals were deprived of ipRGCs, they demonstrated a disorganized biological behavior such as sleep and rest-activity (4).

Degeneration of the retinal ganglion cells (RGCs) and their axons, the retinal nerve fiber layer (RNFL), in the inner retina has been noted in histopathologic studies in the autopsy of Alzheimer's disease. In addition, *in vivo* study of retinal structure using Optical Coherence Tomography (OCT) demonstrated RGC/RNFL loss in not only patients with Alzheimer's disease but also individuals with mild cognitive impairment (MCI) (8–15), and RGC/RNFL thickness was negatively correlated with cognition measured by neuropsychological testing (16–18). Furthermore, intra- and extra-cellular amyloid deposition in the ipRGCs in addition to ipRGC loss were found in postmortem retina in patients with Alzheimer's disease (19).

The ipRGCs are connected to the pretectal olivary nuclei, the pupil regulatory center located in the rostral midbrain (20). The post-illumination pupil response (PIPR), elicited by tailored light stimuli in humans, has been identified as a robust biomarker for ipRGC activity (21, 22). The PIPR correlation with cognition has not been studied. Based on widespread evidence linking RGC loss and central nervous system (CNS) neurodegeneration, we postulate that there could be a potential association between the PIPR and cognitive measures.

Here, we present the first attempt to investigate the association between the PIPR and cognition, one of the most eloquent brain functions, among participants in an established, large epidemiologic cohort.

## Method

### Overview

The study was an ancillary substudy added during the 10-year follow-up Beaver Dam Offspring Study (BOSS), a large longitudinal epidemiologic, multi-sensory study of aging (23, 24). The BOSS used standardized protocols to determine the 10-year incidence of hearing, vision, and olfactory impairments and sensory declines, cognitive function, and the associations of potential risk factors with declines in sensory and cognitive function. The 10-year follow-up BOSS consisted of community-dwelling individuals with age ranging from 27 to 93 years (*n* = 2,466). The majority of the cohort are Caucasians (99.5%), reflecting the demographic characteristics of the parents who were residents of Beaver Dam, WI in 1987–88. In the present study, we invited a convenience sample of 403 BOSS participants to complete pupil recording. The study was approved by the University of Wisconsin Madison Health Sciences Institutional Review Board. Written informed consent was obtained from participants prior to the examination.

The study collected detailed ocular history, including refractive error (non-cycloplegic auto-refraction), self-reported refractive surgery, fundus photo-graded age-related macular degeneration (23) and diabetic retinopathy (25), doctor-diagnosed glaucoma, digital image-graded cataract (26), and self-reported cataract surgery. Relevant history, in particular, blinding eye disease (age-related macular degeneration, glaucoma, and cataract/cataract surgery) and concurrent use of CNS-acting medications (benzodiazepine, antihistamine, and antidepressants), and beta-blockers that may affect pupil reactivity were also collected to use as covariates in the analysis.

### Pupil Recording and Analysis

The binocular infrared pupillometer (DP2000 Human Laboratory Pupillometer, Neur-Optics, Inc., Irvine, CA) consisted of a multi-chromatic binocular/dual camera system that tracks both pupils and stimulates both eyes. The pupillometer recorded the horizontal diameter of the pupil at 30 Hz. The stimulus display presented central stimuli that subtended a visual angle of 50 by 35 degrees at a viewing distance of 39 mm. Participants were kept in a minimally lit exam room for 10 min while receiving instructions prior to pupil recording. The pupil recording commenced with 5 s of darkness. Stimulus protocol consisted of a pair of 1-s bright light stimuli with spectral bands of 640 ± 10 nm (red light) and 467 ± 17 nm (blue light) at a stimulus intensity of 2.0 log lux. Each stimulus presentation was followed by a period of darkness (30 and 60 s following the red and blue light stimulus, respectively) to allow the pupil to settle to baseline. The pupil recording of both eyes was collected when the two eyes were stimulated simultaneously. One trial of pupil recording consisted of two consecutive repeats of the red/blue stimulus pair. The total duration of a trial lasted for approximately 3 min. Two trials of pupil recording were collected for each individual with an interval of 10 min.

**Figure 1:**
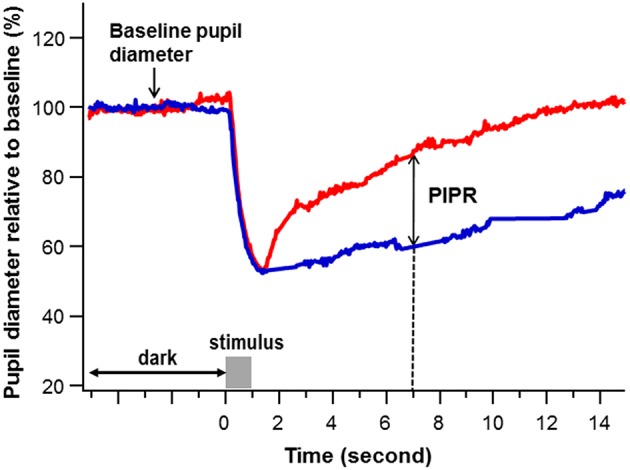
An example of pupil recording. The percent pupil diameter relative to baseline was plotted against time to a 1-s red and blue stimulation at a stimulus intensity of 2.0 log lux (gray bar). The *baseline pupil diameter* was calculated as the average pupil diameter in millimeters over 5 s immediately before the stimulus onset. The *post-illumination pupil response (PIPR)* (double arrow) was calculated as the difference of the pupil contraction amplitude relative to baseline pupil diameter (%) between the blue (blue) and red (red) light stimulation at 6 s after termination of the 1-s light stimulus (The dashed line points the PIPR to 7 s on the x-axis, which is 6 s post stimulus-offset).

Pupil recordings were saved in software-specific raw data files. Using a customized computer software program, pupil metrics were harvested after removal of blinks and recording artifacts. The baseline pupil diameter (BPD) was defined as the mean diameter over the 5-s interval immediately before each stimulus onset. The PIPR was calculated as the difference of the percent pupil contraction amplitude relative to BPD between the blue and red light stimulation at 6 s after termination of the 1-s light stimulus (21, 27, 28) (Figure 1).

### Evaluation of Cognition

Cognitive testing included Trail Making Test (TMT) Parts A and B, Rey Auditory Verbal Learning Test (AVLT), Digit Symbol Substitution Test (DSST), and Verbal Fluency Test (VFT) (F, A, and S). A principal component analyses (PCA) score was created for each participant to summarize cognitive tests together to yield a more sensitive measure of aging changes. The cognitive testing were conducted either during the same visit as the pupil recording or in a separate visit, with the duration ranges from 0.0 to 2.2 years (mean 0.7 years). The BOSS also included co-morbidity data, including diabetes mellitus (doctor-diagnosed or HgA1C > 6.5), obesity [body mass index (BMI) ≥ 30], hypertension (systolic pressure > 140 mmHg or diastolic pressure > 90 mmHg, or doctor-diagnosed or current antihypertensive use), cerebrovascular disease (CVD), doctor-diagnosed thyroid disease, headache/migraine (doctor-diagnosed migraine or experienced severe headaches or migraines over the past 3 months), tobacco and alcohol (any alcohol in the past year) use, and the short form (SF)-12 physical and mental component summary (PCS/MCS) score (29). These co-morbidity data were included as potential covariates for cognition, along with educational attainment.

Dementia was defined as participant report of memory loss affecting functionality and impairment in two or three cognitive domains (executive function: TMT, DSST; Memory: AVLT; Verbal: VFT), an MMSE score <24, or report of doctor diagnosis of AD or dementia. MCI was defined as a participant report of memory difficulties and impairment in at least one of the four cognitive domains.

Since both pupil reactivity and cognitive tests can be affected by alertness and attention, participants were screened with a modified self-reported Morningness-Eveningness questionnaire (30) to control for the circadian phase in which pupil recording and cognitive tests were conducted (31).

### Statistical Analysis

All analyses were conducted using SAS software, version 9.4 (SAS Institute Inc.). Correlation across the pupil measurements was estimated via the Pearson correlation coefficient. Principal component analysis (PCA) with the Factor procedure (Method = Principal with Score option) was used to construct a composite measure (mean of zero and a standard deviation of 1) of the cognitive function (TMT A and B, VFT, AVLT, DSST) test data. The first component was retained, and a PCA score was calculated for the participants as a linear combination of the standardized observed variables.

**Figure 2:**
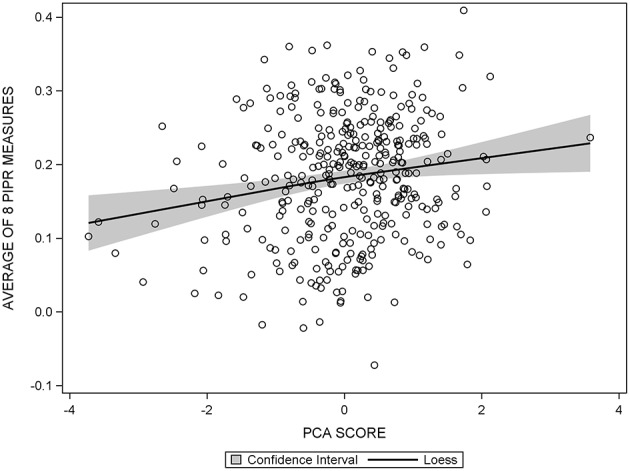
Scatter plot of the average PIPR against PCA score, overlaid with the 95% confidence interval of the loess curve, showing a linear relationship between the average PIPR and PCA score.

The diagnostic panel in the regression output from SAS allowed for assessment of any violations of linear regression using our data. Patterns in the plots of residuals vs. predicted values, *q*-*q* plots, and spread of the residuals were explored. After no violations regarding the assumptions of linear regression were found (Figure 2), the PCA score and individual cognitive function tests were modeled as continuous outcomes in assessing the association with the PIPR using linear regression. Both unadjusted and multivariable linear regression models including age, sex, and education, use of CNS-acting medications and beta-blockers, depression, headache/migraine, cardiovascular disease, ocular disease, SF-12 PCS/MCS, and Morningness-Eveningness status were evaluated.

## Results

A total of 403 BOSS participants (172 male and 231 female) participated in this sub-study with an age range of 33–81 years (mean ± SD, 60.7 ± 9.3). Table 1 describes the demographic features of the participants. The pupil recordings from 377 participants (163 male and 214 female) were included in the analysis, resulting in a data retention rate of 93.5%. A total of 26 subjects were excluded due to excessive artifacts and incomplete pupil recording (due to discomfort from the bright light exposure, three individuals). The demographic features of the 377 included participants were similar to the overall sample (see Supplementary Table 1). The cognitive assessment identified MCI in 14 (3.7%) and dementia in 2 (0.5%) participants, with the rest of the 361 participants being normal (95.8%). The mean and median of the cognitive function tests (PCA score and categorized) were demonstrated in Supplementary Table 2.

**Table 1:** Participant characteristics.

**Characteristic (*n* = 403)**	**Mean**	**SD**
Age	60.7	9.3
BMI (kg/m^2^)	31.0	7.0
SF-12 PCS	47.9	9.5
SF-12 MCS	53.2	7.5
**Characteristic**	***N***	**Percent (%)**
Sex		
Female	231	57.3
Male	172	42.7
Education (years)		
0–12	127	31.7
13–15	162	40.4
16+	112	27.9
Systemic comorbidity		
CVD	42	10.6
Diabetes	46	11.4
Hypertension	205	51.0
Headache	42	10.4
Migraine	60	14.9
Thyroid disease	80	19.9
Smoking status		
Never	204	51.8
Past	134	34.0
Current	56	14.2
Alcohol use in the past year	345	86.3
Systemic medication		
Antihistamine	67	17.8
Benzodiazepine	26	6.9
Beta-blockers	69	18.3
Antidepressants	66	17.5
Ocular comorbidity		
Refractive error (right eye)		
Myopia	123	30.8
Emmetropia	166	41.6
Hyperopia	110	27.6
Glaucoma	11	2.7
Cataract	39	12.4
Cataract surgery	32	7.9
ARMD	16	4.4
Diabetic retinopathy	15	4.1
Morningness-eveningness type		
Morningness	253	63.0
Eveningness or neither	149	37.0

*PCS, physical component summary; MCS, mental component summary; SD, standard deviation; BMI, body mass index; CVD, cardiovascular disease; ARMD, age-related macular degeneration*.

The PIPR was found to be highly correlated between the two eyes, the repeated runs within a trial, and the two trials by linear regression and Bland-Altman plot (see Supplementary Table 3). The mean PIPRs across all available PIPRs for individual participants were used for subsequent analysis of the PIPR vs. cognitive relationship. A minimum of four PIPRs was required for each participant to be included in the analysis.

The BPD ranged from 2.5 to 7.7 mm (mean ± SD: 5.3 ± 0.9) (right eye, trial 1). The BPD appeared to be stable within a trial of recording (estimated bivariate correlations, Pearson Correlation Coefficients > 0.92). There was a significant reduction of the BPD by 0.21 mm for every 5-year increase in age (95% CI: −0.25, −0.17; *p* < 0.001). There also appeared to be an age-related decrease in the mean PIPR by 0.01 for every 5-year increase in age (95% CI: −0.02, −0.01). However, the PIPR vs. age correlation was no longer significant after adjusting for the BPD.

The mean PCA cognitive score increased by 0.20 (95% CI: 0.08, 0.32) for every 0.1 unit increase in the mean PIPR (linear regression, *p* < 0.01); the association remained significant (adjusted estimates 0.13; 95% CI: 0.01, 0.25, *p* = 0.04) after adjusting for age, sex, BMI, education, medication (antihistamine, benzodiazepine, antidepressants, and beta-blockers), depression, headache/migraine, cardiovascular disease, ocular disease (glaucoma, cataract/cataract surgery, and age-related macular degeneration), SF12 PCS/MCS, and Morningness-Eveningness status (Table 2). When breaking down into individual cognitive measures, TMT A and B but not the other cognitive tests remained significantly associated with the mean PIPR.

**Table 2:** The full model with the adjustment for variables to assess the association between the PIPR and PCA summary score of the five cognitive measures (*n* = 377).

**Variable**	**B coefficient (95% CI)**	***P-*value**
Age at pupillometry exam (in 5 year increments)	−0.15 (−0.21, −0.09)	** <0.0001**
Sex (male gender)	−0.72 (−0.93, −0.51)	** <0.0001**
BMI (kg/m^2^)	−0.004 (−0.019, 0.011)	0.63
College education (16+ years)	0.49 (0.26, 0.72)	** <0.0001**
SF-12 PCS	0.0005 (−0.01, 0.01)	0.94
SF-12 MCS	0.007 (−0.01, 0.02)	0.41
Morning vs. evening/neither type	−0.005 (−0.22, 0.21)	0.96
Medication use		
Antihistamine	0.11 (−0.14, 0.37)	0.39
Benzodiazepine	0.27 (−0.15, 0.69)	0.21
Antidepressants	−0.06 (−0.32, 0.20)	0.66
Beta-blockers	−0.05 (−0.35, 0.24)	0.71
Cardiovascular disease	0.30 (−0.07, 0.67)	0.11
Depression	0.30 (−0.15, 0.75)	0.19
Headache/migraine	0.14 (−0.11, 0.40)	0.28
Glaucoma	−0.11 (−0.69, 0.48)	0.72
Any cataract, worse eye	−0.14 (−0.47, 0.18)	0.39
Ever had cataract operation	0.004 (−0.52, 0.52)	0.99
ARMD (worse eye)	−0.41 (−0.89, 0.06)	0.09
Average PIPR (in 0.10 unit increments)	0.13 (0.008, 0.25)	**0.04**

*The bold values show statistically significant correlation*.

## Discussion

In this cross-sectional study, we found a modest correlation between the PIPR and PCA summary score of four cognitive function tests among the participants in a subgroup of the BOSS cohort. The results suggest that longitudinal studies to investigate the PIPR as a potential predictor for cognition, one of the most important measures of brain aging, are warranted.

Our study was not designed to evaluate the causal relationship between the PIPR and cognition. The current evidence suggests a different trajectory of the PIPR and cognitive change during aging, with preservation of the PIPR up to age 70, whereas cognitive decline can take place decades earlier (32, 33). The ipRGCs connect to widespread brain areas besides the suprachiasmatic nuclei of the thalamus and pretectal olivary nuclei of the midbrain, including the lateral geniculate nucleus, ventral subparaventricular zone, the ventrolateral preoptic nucleus, and intergeniculate leaflet (34). However, these areas are not generally considered to be directly involved in cognitive function. A possible explanation of the observed association between the PIPR and cognition could be due to a unified CNS neurodegenerative process equally affecting the ipRGCs and brain networks involved in cognition, given that there has been ample evidence showing an overall retinal ganglion cell loss in Alzheimer's disease (18, 19, 35).

The individual cognitive tests used in this study measure different aspects of cognition, with the TMT A and B for visuomotor speed and set-shifting, AVLT for rote verbal learning and memory, DSST for processing speed, and VFT for language and executive functioning. When breaking down into individual cognitive tests, TMT A and B, but not the other cognitive measures show a significant correlation with the PIPR. Such a finding is not surprising as the TMT is a test of attention requiring quick visual scanning and object recognition, and both pupil reactivity and overall cognitive performance can be influenced by participants' attention (36, 37). Interestingly, the DSST also requires quick visual scanning and object recognition, but we did not find a direct relationship between it and PIPR. This suggests that a skill specific to TMT A and B may be driving this connection. In this study, we included the Morningness-Eveningness questionnaire to assess the impact of circadian phase on the outcome measures. The circadian phase during which cognitive testing is performed can impact cognition; a morning person will perform better in the morning when they are readily awake thus able to better concentrate and attend to the tests. In addition, the PIPR in humans has demonstrated a circadian variation over a 24-h cycle (31). In our study, the majority of the participants are morning type and adjusting for Morningness-Eveningness status did not change the outcomes. Further study including attention parameters may provide useful information in assessing whether the correlation between the PIPR and cognition demonstrated in our study indeed relates to attention.

Identifying biomarkers of brain neuronal activity has been an ongoing effort in the pursuit of a better understanding of brain aging. A few biomarkers have been found to link amyloid-beta, the hallmark of Alzheimer's disease, to early cognitive changes detected in cognitive tests. However, these biomarkers, including cerebrospinal fluid (CSF) and positron emission tomography (PET) amyloid imaging markers, functional magnetic resonance imaging (fMRI), CSF tau, and MRI brain volume quantification (38–40), are either proxies of the brain neuronal activity as they do not directly test neuronal function or costly, thus limiting their widespread use. The PIPR offers a unique benchmark by quickly and directly measuring the physiologic function of the ipRGCs, the brain neurons involved in non-image-forming function. Results of this study suggest that the PIPR is related to cognition, although future studies are needed to better understand the nature of this relationship. The PIPR is less invasive and costly than the currently available proxy measurements and has the promise to serve as a screening tool for cognitive impairment. However, we should still be aware that the PIPR does not reflect the function of the brain neurons directly involved in cognition.

We acknowledge several limitations in the present study. First, our study used a convenience sample of the BOSS which could be subject to selection bias. Comparison of multiple variables between our sample and the overall BOSS cohort demonstrated similar demographic features (Supplementary Table 1), suggesting that our cohort is a fair representation of the community-dwelling middle-aged and older adult population of the upper Midwest. However, the predominance of non-Hispanic whites in this cohort may limit the generalizability of the study results to other racial groups. Second, the study results should be interpreted with caution given the cross-sectional study design, where cognitive impairment can be confounded by cohort differences in education even after adjusting for age, sex, and education. Third, it is worthwhile to point out the heterogeneity within our cohort, especially where ocular comorbidity is concerned. The small proportion of the diseased eyes may have contributed to the variability of the PIPR and confounded the interpretation of PIPR vs. cogntion correlation. To address this concern, we did a sensitivity analysis excluding those who had ocular diseases (fundus photo-graded age-related macular degeneration and diabetic retinopathy, doctor-diagnosed glaucoma, digital image-graded cataract, and self-reported blinding eye disease). The analysis showed similar results. Incorporating additional structural and functional evaluation of RGCs in future studies would provide valuable information in clarifying the interrelation between the PIPR, age-related ocular disease, and cognition. Lastly, the present study did not allow us to evaluate the impact of the circadian phase on PIPR/cognition correlation. The experimental study of Munch et al. demonstrated a circadian oscillation of the PIPR in humans (31). The present study did not evaluate participants' circadian rhythm; in fact, evaluating circadian rhythm would be challenging in field study. We did perform an analysis where we incorporated the time of the pupil recordings in the model; the analysis showed no significant impact of the recording time on the results. Furthermore, the majority of our study cohort were morning type and all the pupil recordings were collected during the daytime (half from 8 to 11). Therefore, it would be unlikely that the time of the pupil recording would impose a significant impact on PIPR/cognition correlation in this study.

## Conclusions

In this cross-sectional study, we demonstrated the feasibility of collecting the PIPR in an epidemiologic cohort and found a modest association between the PIPR and PCA summary score of four cognitive function tests. A longitudinal study is warranted to evaluate the role of the PIPR in predicting cognitive change among community-dwelling middle aged and older adults across all racial groups.

## Ethics Statement

All subjects gave written informed consent in accordance with the Declaration of Helsinki. The protocol was approved by the University of Wisconsin Madison Health Sciences Institutional Review Board.

## Author Contributions

YC and KC contributed to conception and design of the study. AAP organized the database and performed the statistical analysis. YC wrote the first draft of the manuscript. All authors contributed to manuscript revision, read and approved the submitted version.

### Conflict of Interest Statement

The authors declare that the research was conducted in the absence of any commercial or financial relationships that could be construed as a potential conflict of interest.
