# Supplementary material for: The Post-illumination Pupil Response (PIPR) Is Associated With Cognitive Function in an Epidemiologic Cohort Study
Source: Front Neurol. 2019 Jun 26;10:682. doi: 10.3389/fneur.2019.00682 (PMC6607919; doi:10.3389/fneur.2019.00682)
Supplement: Supplementary file 1 [file Table_1.pdf]

Supplementary Table 1. Comparison of baseline participant characteristics between our sample and the rest of the BOSS cohort

Characteristic	Our sample (n=403)		BOSS (n=2063)		P-value
	n	%	n	%	
Sex					
Male	172	42.7	930	45.1	0.38
Female	231	57.3	1133	54.9	
Education (years)					
0-12	127	31.7	494	24.1	<0.001
13-15	162	40.4	689	33.7	
16+	112	27.9	864	42.2	
Systemic comorbidity					
CVD	42	10.6	245	12.0	0.41
Diabetes	46	11.4	244	11.9	0.79
Hypertension	205	51.0	913	53.2	0.42
Headache	42	10.4	219	10.7	0.88
Migraine	60	14.9	311	15.2	0.89
Thyroid disease	80	19.9	285	14.0	0.002
Smoking Status					
Never	204	51.8	1208	59.3	0.004
Past	134	34.0	634	31.1	
Current	56	14.2	195	9.6	
Alcohol use in the past year	345	86.3	1772	86.8	0.76
Systemic medications					
Antihistamine	67	17.8	254	16.3	0.48
Benzodiazepine	26	6.9	71	4.6	0.06
Beta-blockers	69	18.3	233	14.9	0.10
Antidepressants	66	17.5	295	18.9	0.54
Ocular comorbidity					
Refractive error					
Myopia	123	30.8	536	34.7	0.03
Emmetropia	166	41.6	678	43.9	
Hyperopia	110	27.8	330	21.4	
Glaucoma	11	2.7	60	2.9	0.84
Cataract	39	12.4	187	10.5	0.32
Cataract surgery	32	7.9	162	7.9	0.99
ARMD	16	4.4	83	4.1	0.77
Diabetic retinopathy	15	4.1	131	6.4	0.09
	Mean	SD	Mean	SD	
Age (years)	60.2	9.4	58.4	9.7	0.35
BMI (kg/m^2)	31.0	7.0	31.1	6.8	0.80
SF-12 MCS	53.2	7.5	52.5	8.1	0.06
SF-12 PCS	47.9	9.5	49.1	9.1	0.01

Supplementary Table 2. The range, mean, and median of the cognitive function tests for each modality and PCA score.

Cognitive function test	N	Minimum	Maximum	Mean	SD	Median
TMT A	374	7.0	93.0	29.6	11.2	27.0
TMT B	373	11.0	301.0	72.5	35.7	65.0
VFT	371	10.0	92.0	41.0	12.0	41.0
AVLT	367	0.0	15.0	7.1	2.8	7.0
DSST	374	23.0	93.0	55.2	12.0	56.0
PCA score	362	-3.7	3.6	0.0	1.0	0.1

Supplementary Table 3. Correlations among the 8 PIPR measurements (2 Trials, 2 eyes, 2 runs). The numbers represent the Pearson correlation coefficient values.

	Tr1_R_Run1	Tr1_R_Run2	Tr1_L_Run1	Tr1_L_Run2	Tr2_R_Run1	Tr2_R_Run2	Tr2_L_Run1	Tr2_L_Run2
Tr1_R_Run1	1.00							
Tr1_R_Run2	0.60	1.00						
Tr1_L_Run1	0.92	0.63	1.00					
Tr1_L_Run2	0.53	0.90	0.63	1.00				
Tr2_R_Run1	0.54	0.54	0.57	0.56	1.00			
Tr2_R_Run2	0.48	0.59	0.53	0.59	0.55	1.00		
Tr2_L_Run1	0.50	0.49	0.55	0.53	0.85	0.53	1.00	
Tr2_L_Run2	0.54	0.59	0.57	0.62	0.57	0.90	0.54	1.00

Tr1: Trial 1, Tr2: Trial 2, R: Right eye, L: Left eye
